# Non-Specific Manipulation of Gammarid Behaviour by *P. minutus* Parasite Enhances Their Predation by Definitive Bird Hosts

**DOI:** 10.1371/journal.pone.0101684

**Published:** 2014-07-07

**Authors:** Lisa Jacquin, Quentin Mori, Mickaël Pause, Mélanie Steffen, Vincent Medoc

**Affiliations:** 1 Institute of Ecology and Environmental Sciences (iEES, UPMC-CNRS) UMR 7618, Université Pierre et Marie Curie, Paris, France; 2 McGill University, Department of Biology & Redpath Museum, Montréal, Québec, Canada; Swansea University, United Kingdom

## Abstract

Trophically-transmitted parasites often change the phenotype of their intermediate hosts in ways that increase their vulnerability to definitive hosts, hence favouring transmission. As a “collateral damage”, manipulated hosts can also become easy prey for non-host predators that are dead ends for the parasite, and which are supposed to play no role in transmission strategies. Interestingly, infection with the acanthocephalan parasite *Polymorphus minutus* has been shown to reduce the vulnerability of its gammarid intermediate hosts to non-host predators, whose presence triggered the behavioural alterations expected to favour trophic transmission to bird definitive hosts. Whilst the behavioural response of infected gammarids to the presence of definitive hosts remains to be investigated, this suggests that trophic transmission might be promoted by non-host predation risk. We conducted microcosm experiments to test whether the behaviour of *P. minutus*-infected gammarids was specific to the type of predator (i.e. mallard as definitive host and fish as non-host), and mesocosm experiments to test whether trophic transmission to bird hosts was influenced by non-host predation risk. Based on the behaviours we investigated (predator avoidance, activity, geotaxis, conspecific attraction), we found no evidence for a specific fine-tuned response in infected gammarids, which behaved similarly whatever the type of predator (mallard or fish). During predation tests, fish predation risk did not influence the differential predation of mallards that over-consumed infected gammarids compared to uninfected individuals. Overall, our results bring support for a less sophisticated scenario of manipulation than previously expected, combining chronic behavioural alterations with phasic behavioural alterations triggered by the chemical and physical cues coming from any type of predator. Given the wide dispersal range of waterbirds (the definitive hosts of *P. minutus*), such a manipulation whose efficiency does not depend on the biotic context is likely to facilitate its trophic transmission in a wide range of aquatic environments.

## Introduction

Trophically-transmitted parasites display complex life cycles relying on predation events between successive hosts. They often trigger alterations of behaviour, physiology and/or appearance in their intermediate hosts [1], which traditionally have been interpreted by three different hypotheses. First, parasite-induced alterations can be side effects of infection that have no adaptive value. Second, they can be compensatory responses of hosts to reduce the detrimental effects of parasites, with the consequence of enhancing predation and benefiting the parasite [2]. Third, alterations can result from an adaptive manipulation of host phenotype that enhances parasite's trophic transmission from the intermediate host (where the parasite develops from one larval stage to the next) to the definitive host (where the parasite matures and reproduces sexually), which is often called “predation enhancement” [3], [4].

But manipulation can also result in “predation suppression” where trophically-transmitted parasites act as “bodyguards”, protecting their intermediate host from the predators that can jeopardize parasite's survival (see [5] for a review). Such protective manipulation typically occurs when the parasite has not yet reached the infective stage (e.g. [6]). For instance, *Anopheles* mosquitoes infected with the non-infective stage of the malaria parasite *Plasmodium* spp. show decreased feeding persistence that could limit mortality associated with blood feeding [7]. Similarly, crustacean gammarids parasitized with the non-infective stage (acanthella) of the acanthocephalan parasite *Pomphorhynchus laevis* show increased antipredatory behaviour (increased refuge use) that limits predation by fish [8]. In those cases, infected hosts show a shift from predation suppression to predation enhancement once the parasite becomes infective, which matches the parasite's interest in terms of transmission.

In other cases, both predation enhancement and predation suppression occur at the same time in the presence of infective stages of the parasite. For instance, gammarids infected by the bird acanthocephalan *Polymorphus minutus* displayed both risky and protective behaviours simultaneously. Compared to uninfected *Gammarus roeseli*, infected individuals display a faster escape from the non-host predatory invertebrate *Dikerogammarus villosus* [9], and a lower activity and increased refuge use that reduce predation by fish non-hosts [10]. At the same time, *P. minutus*-infected gammarids preferentially locate at the air-water interface (geotaxis reversion) [11], [12], which is likely to facilitate predation by waterbird definitive hosts, although this has never been experimentally tested yet.

Protective behavioural alterations against non hosts raise the question of specificity in host manipulation since they allow the overall strategy to better target suitable next hosts. A manipulation is considered specific when it increases next-host predation more than non-host predation [13], [14]. This might be achieved by increasing the vulnerability of intermediate hosts to predation by final hosts, decreasing their vulnerability to predation by non hosts, or both [15]. In the third case, the manipulation shows a high degree of specificity through the combination of predation enhancement and predation suppression [16]. Under the assumption of a highly specific manipulation, it is expected that manipulated intermediate hosts behave differently depending on whether the predator is a definitive host or a non host.

Although theoretical models suggest that even non-specific manipulations are adaptive [17], [18], the authors acknowledge that predation enhancement is always most favourable when it specifically targets the right host [18]. To date, examples of non-specific manipulation outnumber those suggesting some degree of specificity [13], [17], [19], [20], which also comes from the fact that for long, predation enhancement received much more attention than predation suppression. This stresses the need to focus on predation suppression as well as predation enhancement, and to take into account the biotic context in which manipulation occurs (e.g. variable non-host predation risk) so as to better understand the benefits of specificity in transmission strategies.

On the one hand, with a parsimonious view, predation enhancement and predation suppression of a specific manipulation could share the same origin and both result from the same parasite-induced alteration. The apparent reduced vulnerability to non-host predators would be the fortunate consequence (a kind of beneficial side effect) of a parasite-induced alteration that actually promotes definitive-host predation. For instance, in the gammarid - *P. minutus* system, when manipulated gammarids locate at the air-water interface, they are *de facto* out of reach for benthic fish non-hosts (see [21], [22]). Similarly, staying motionless at the water surface may increase the probability of ingestion by waterbirds and by chance decrease the attractiveness to visually-feeding non hosts such as fish. Under this assumption, we could expect no effect of the biotic context (i.e. the presence of non hosts) on gammarids' behaviour and transmission to definitive hosts.

On the other hand, with a more “adaptationist” view, predation enhancement and predation suppression could rely on distinct parasite-induced changes selected for in an independent way. They would be distinct dimensions of a multidimensional manipulation (i.e. when infection with a single parasite changes more than one trait in host phenotype, see [15], [23], [24]), the former favouring final-host predation and the latter reducing the cost of non-host predation [16]. Interestingly, geotaxis reversion, the alteration expected to facilitate trophic transmission in *Polymorphus minutus*, was found to be triggered by the presence of non-host predators [10], [21]. This suggests that non-host predation risk may positively influence trophic transmission. Under this assumption, the biotic context in which manipulation occurs could play a crucial role in the probability of transmission to final hosts.

To address these hypotheses, we tested whether the biotic context (i.e. the presence of non-host predators) influenced the behavioural alterations induced by *P. minutus* in its gammarid intermediate hosts and investigated the consequences in terms of transmission to bird definitive hosts, using a combination of experimental approaches in microcosms and mesocosms. In microcosm experiments, we investigated the effect of the olfactory context on several behavioural traits linked to predation risk (avoidance of predation cue, activity, geotaxis and conspecific attraction) in water scented with either fish (non host) or waterbirds (definitive host). In mesocosm experiments, we followed the vertical distribution of both uninfected and infected gammarids after successive introductions of fish and waterbird to the device and tested their effects on predation risk by waterbirds. Under the parsimonious hypothesis, we expected infected gammarids to behave similarly with fish or waterbird predator cues. We also expected the presence of non hosts in the mesocosms to have no effect on the probability of being eaten by birds. Under the adaptationist hypothesis, we expected the behavioural response of manipulated amphipods to be fine-tuned to the type of predator (for instance, avoidance of fish cues but attraction towards waterbird cues), and a higher trophic facilitation to waterbirds in the presence of fish non-hosts.

## Methods

### Model species, sampling and housing conditions

All experiments were performed at the CEREEP field station (Centre de Recherche en Ecologie Expérimentale et Prédictive, St-Pierre-lès-Nemours, France). Domestic mallards *Anas platyrhynchos* (10 females and four males), known to be definitive hosts of *Polymorphus minutus*, were housed in a permanent aviary (10 m long x 4 m wide x 3 m high) equipped with an artificial pond, shelters, and fed *ad libitum* with a mix of maize, wheat and peas. Gammarids (*Echinogammarus berilloni*) and fish (sticklebacks *Gasterosteus aculeatus* and minnows *Phoxinus phoxinus*) were caught from the Lunain river at Nonville (48°17′31.44″N- 2°47′8.68″E, France). Gammarids and sticklebacks were sampled using a hand net (500 µm mesh) and minnows with a battery-powered portable electrofishing gear (Hans Grassl IG600 type, Aquaculture, France). In the Lunain river, mallards are present and *E. berilloni* coexists with *Gammarus pulex* but *P. minutus* is found in the former only (mean prevalence near the substratum  = 1.88%; near the surface  = 13.27%, unpublished data). The carotenoid-based colouration of *P. minutus* cystacanths makes them appear as a bright-orange spot through the translucent cuticle of infected gammarids, which distinguishes them from uninfected individuals. To standardize gammarid size, we selected intermediate-sized individuals (10±1 mm in total length, from the tip of the rostrum to the base of the telson) and excluded gravid females. To avoid the confounding effects associated with multiple infections, gammarids harbouring several cystacanths of *P. minutus* or infected with other symptomatic parasites such as the fish acanthocephalan *Pomphorhynchus laevis* and muscle wasting microsporidians were excluded.

In the laboratory, gammarids were kept in 10-L tanks (30 cm long x 20 cm wide x 20 cm high) filled with filtered water from the Lunain river at a maximum density of 20 ind./L and fed at satiation with conditioned elm leaves. Sticklebacks and minnows were kept in 65 and 150-L tanks, respectively, at a maximum density of three ind./L, and fed every day with commercial flake food. In each housing unit, oxygen was supplied by an air pump and macrophytes and stones from the sampling site were added to provide shelters and limit stress. The tanks were placed in a thermoregulated room to ensure a stable temperature (around 15°C) under natural daylight conditions. Experiments took place in the same thermoregulated room to standardize conditions between housing and experiments.

### Ethics statement

This study was carried out in strict accordance with the recommendations of the European Convention for the Protection of Vertebrate Animals used for Experimental and Other Scientific Purposes (Appendix B). Captures in the Lunain river were carried under the permission of the owner of the land. Captures, experiments and protocols did not involve endangered or protected species and were approved by the “Direction Départementale des Services Vétérinaires de Seine-et-Marne” (Permit Number N°A77-431-1).

### Reaction to predation cue and activity in microcosms

**Figure 1:**
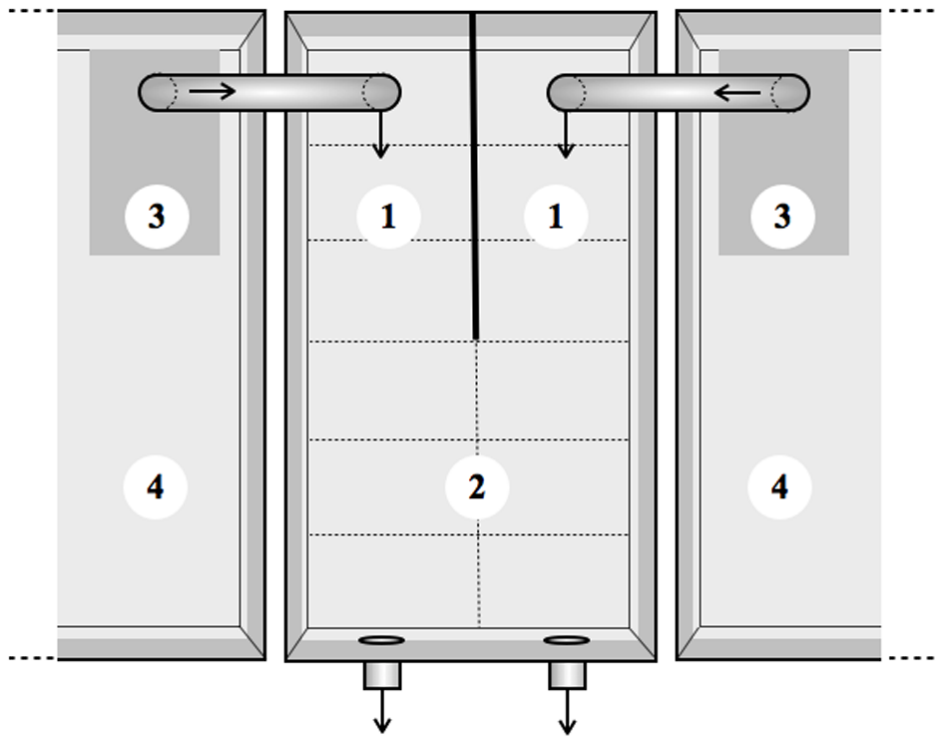
Schematic top view of the olfactometer used to investigate how *Polymorphus minutus*-infected and uninfected *Echinogammarus berilloni* react to predation cues. The olfactometer consisted of two arms (1) receiving an inflow of either scented water (fish or mallard odour) or control water, and a mixing zone (2) where the gammarid was introduced at the beginning of the experiment. Water inflows were provided by water pumps (3) connected to the plastic tanks filled with either scented or control water (4). The olfactometer was divided into equal-sized areas to measure activity (dashed lines).

We used an olfactometer to test whether *P. minutus*-infected gammarids were attracted to, repulsed by or indifferent to the scent of either definitive host or non-host predators (Fig. 1). The olfactometer consisted of a glass aquarium (35 cm long ×20 cm wide ×25 cm high) divided into three zones: a scented arm receiving an inflow of scented water (fish or mallard odour, see below), a control arm receiving filtered water from the sampling site, and a mixing zone where water was mixed. For each arm, water inflow was provided by a water pump connected to a 50-L plastic tank. To record gammarid's activity, each zone was virtually divided into distinct areas using a marker on the outside face of the bottom. Preliminary tests using dyes enabled us to set a flow rate of 180 mL/min (4-cm water height), so that inflowing water reached the point of confluence after 20 seconds. Such a flow rate prevented mixing of water inside one of the two arms and allowed a complete mix of water in the mixing zone after 60 seconds. The olfactometer worked as an open system and mixed water was not recirculated (Fig. 1). Fluorescent tubes mounted 90 cm above the olfactometer provided homogeneous light conditions (7±1 Wm at the water surface, measured with an ALMENO 2890-9 pyranometer).

Gammarids (N = 70 per infection status) were tested individually and twice: once with fish odour and once with mallard odour in the scented arm, the order and side of the scented arm being changed every 10 gammarids. This allowed us to control for order effect and side preference. To remove predation cue when we switched between fish and mallard odours, the pumps and the tanks were rinsed with ethanol and tap water. The tested gammarid was introduced in a perforated tube in the middle of the mixing zone. After a five-min acclimatization period, the tube was gently removed to release the gammarid and its behaviour was recorded during five minutes using the Jwatcher software (www.jwatcher.ucla.edu). We recorded the proportion of time spent in the scented arm and its activity corrected by time, as the total number of zones crossed in the whole olfactometer or within one arm divided by the time spent within the corresponding arm. Two infected individuals spent more than two minutes motionless and were not considered in the analysis because they did not spend enough time in any arm to provide reliable estimation of relative odour preference [25], [26]. Water was changed and the tanks rinsed with ethanol and tap water after each test to remove the olfactory cues. After the experiment, gammarids were kept individually overnight in Petri dishes in the housing room and tested for geotaxis and conspecific attraction the day after.

### Geotaxis and conspecific attraction in microcosms

**Figure 2:**
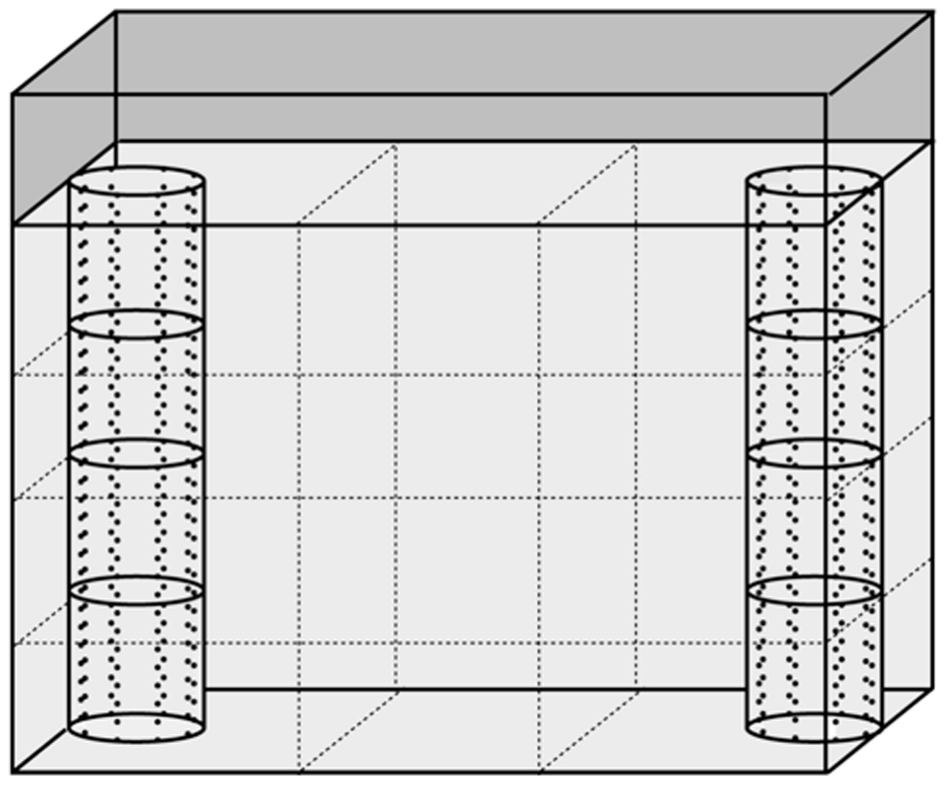
Schematic side view of one of the three aquariums used to investigate geotaxis and conspecific attraction in *Polymorphus minutus*-infected and uninfected *Echinogammarus berilloni*. Two transparent plastic tubes, perforated and divided into four equivalent compartments, were placed at each end close to the inside. One of the tubes was filled with gammarids while the other remained empty. The aquarium was divided (dashed lines) into four equal-sized areas along its height and three equal-sized areas along its length to measure geotaxis and conspecific attraction, respectively.

Three 10-L aquariums (30 cm long ×10 cm wide ×25 cm high) were filled with filtered water from the sampling site and equipped with two transparent plastic tubes (5 cm diameter, 20 cm height), placed at each end close to the inside (Fig. 2). In each tank, one tube was filled with 20 uninfected *E. berilloni* (from 9 to 12 mm in total length) while the other remained empty. The tubes were perforated to allow water and chemical cue exchange, and divided into four vertical compartments of five cm each. Five gammarids were randomly placed in the four compartments to obtain a homogeneous distribution along the water column and hence avoid any effect of conspecific attraction on the vertical distribution of the tested gammarid. To measure geotaxis, we virtually divided the water column into four equal-sized areas that matched the subdivision of the tubes. Similarly, to measure conspecific attraction, each aquarium was divided along its length into three equal-sized areas of 10 cm^2^. The middle area served as neutral zone whereas the outer areas served as choice zones with (full tube) or without conspecifics (empty tube). Of the three tanks, a first one was filled with water from the sampling site previously aerated for 24 h to remove any chemical signal, a second one with fish-scented water, and the last with waterbird-scented water (see below). As for the previous experiment (activity and reaction to predation cue), fluorescent tubes mounted 90 cm above the olfactometer provided homogeneous light conditions (7±1 Wm at the water surface, measured with an ALMENO 2890-9 pyranometer).

All gammarids successively experienced the three types of water in each scented aquarium, the order being changed every 10 individuals. Infected and uninfected gammarids were tested alternatively. The tested gammarid was introduced in a perforated opaque tube in the middle of the neutral zone. After a five-min acclimatization period, the tube was gently removed and the vertical and horizontal positions of the gammarid were recorded every 30 s over five min using the JWatcher software. At each record (10 per test), geotaxis and aggregation scores were assigned depending on the location. The geotaxis score was “1” in the bottom area and increased by one going towards the water surface to reach the maximum value of “4” in the top area. The aggregation score was “0” for the neutral area, “−1” for the choice area without gammarids and “+1” for the choice area with gammarids. At the end of each test, summed scores for geotaxis ranged from “10” (always in the bottom area) to “40” (always in the top area), and for aggregation from “−10” (always in the choice area without gammarids) to “10” (always in the choice area with conspecifics). Every 10 gammarids, the side of the empty tube was changed to avoid any confounding effect of spatial preference, water was changed and the aquariums were rinsed with tap water and diluted ethanol to remove any olfactory cue. The gammarids that stayed motionless during the whole experiment (12 out of the 70 infected gammarids and 2 out of the 70 uninfected gammarids) were excluded from the analyses, because we could not determine whether their location in the water column was due to altered geotaxis or to poor health status. Accordingly, these individuals died few hours later.

### Scented water

To obtain fish-scented water, we placed five intermediate-sized sticklebacks of approximately 6 g together with 45 uninfected *E. berillloni* in a plastic tank filled with 35 L of aerated and filtered water from the sampling site. This represented a biomass of stickleback of 0.85 g/L. After a 24 h exposure, all the gammarids were consumed, representing approximately 1.3 predation events per litre (see [10], [27]). This process was expected to provide a predation signal close to the one observed under natural conditions, because it includes both the fish odour and the chemical cues released by injured gammarids [28]. To obtain waterbird-scented water, we placed one mallard of approximately 950 g in a plastic tank filled with 15 L of aerated water from the sampling site during 45 minutes. A pilot experiment showed that the resulting scented water induces a significant change in gammarids' activity, measured as the number of times the tested individual crossed a virtual line dividing a Petri dish into two equal parts (paired Wilcoxon test between control and mallard-scented water: N = 20, V = 152, P = 0.02).

### Vertical distribution in mesocosms

**Figure 3:**
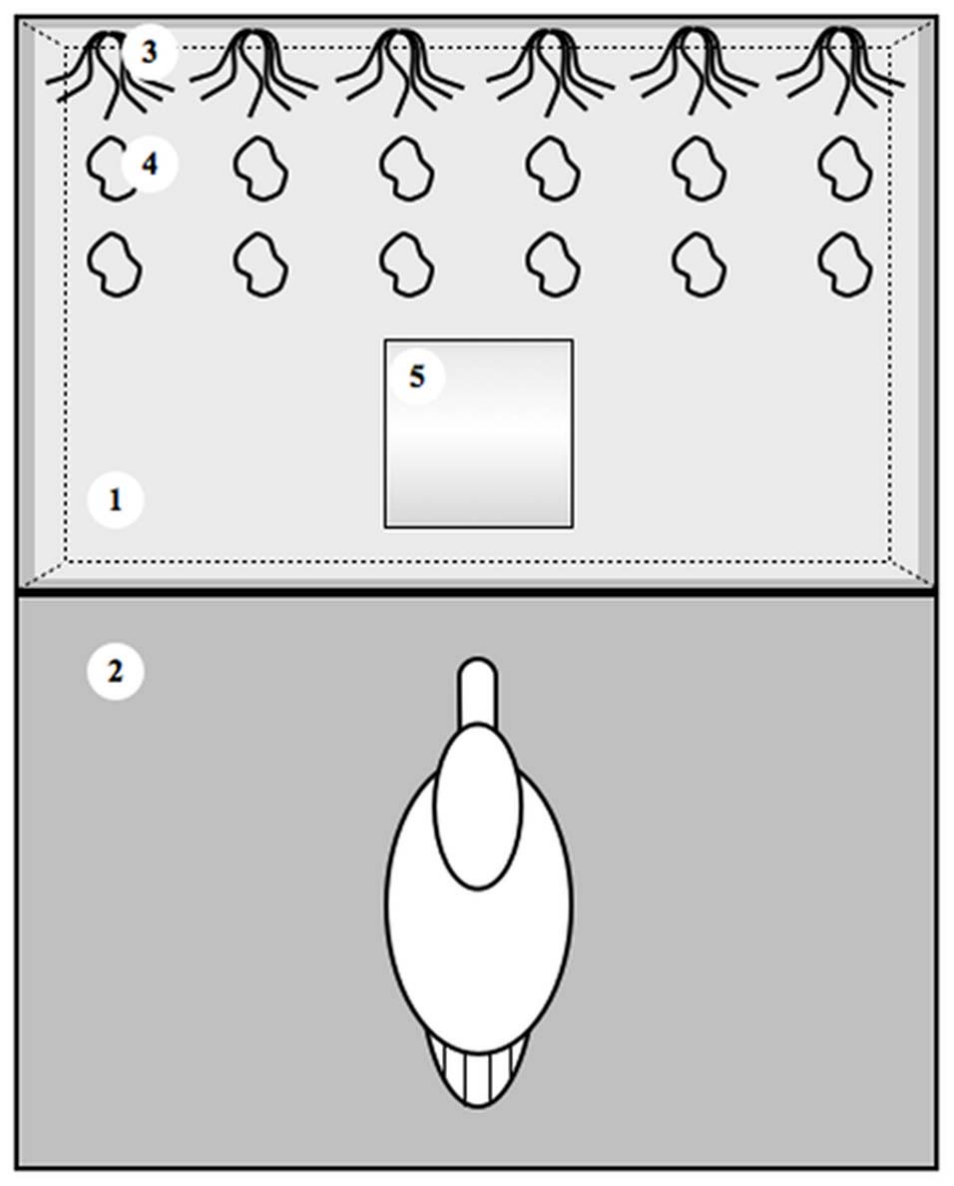
Schematic top view of one of the 12 mesocosms used to investigate how changes in the biotic context influence the vertical distribution of *Echinogammarus berilloni* either uninfected or infected with *Polymorphus minutus*, and their vulnerability to mallard predation. Each mesocosm included a water tank (1) with an access platform (2) and equipped with refugia at the air-water interface (3) and on the bottom (4). During the course of the experiment, a plastic tube (5) either empty of filled with fish was introduced into the water tank to mimic non-host predation risk (see text for details).

We designed 12 mesocosms so as to mimic a riverbank, each including a black plastic tank (80 cm long ×63 cm wide ×38 cm high) and an access platform of equivalent area (Fig. 3). Tanks were filled with 50 L of filtered water from the sampling site and 80 L of tap water, representing a water column of 35 cm. We used a mix of stream water and tap water to reduce the volume of stream water needed. We provided six refugia at the air-water interface to mimic the natural plants where waterbirds forage, and where *P. minutus*-infected gammarids are found naturally. They consisted of 12 six-cm long strings of dark-green wool attached by one end, and were equally distributed along one length of the tank. The wool was boiled and rinsed several times to remove any compounds that may be repellent to gammarids. We did not use natural plants that can also serve as food source, hence representing a confounding effect. As benthic refugia, 12 stones from the sampling site (7 to 10 cm in diameter), whose perilithon was removed with a metallic brush, were placed on the bottom. As food source, equivalent amounts of conditioned *Alder* leaves were distributed between the stones and between the wool strings.

At the beginning of the experiment, 50 *P. minutus-*infected and 100 uninfected *E. berilloni* were introduced in each tank. After 24 h, we mimicked non-host predation risk in six randomly chosen mesocosms by adding a plastic tube (25 cm long and 21 cm diameter) filled with 12 fish (2 sticklebacks and 10 minnows) and 65 uninfected *E. berilloni* so as to have approximately 1.3 predation events per litre. The plastic tubes were closed at each end with a net (500 µm mesh) to allow chemical cue exchange with surrounding water. The six other mesocosms received an empty tube and served as control group. This allowed us to disentangle the respective influences of the physical disturbance due to the introduction of the tube and the chemical stimulation linked to fish predation on gammarids' behaviour. At 115 h after the introduction of gammarids, we mimicked definitive-host predation risk by introducing into each mesocosm a mallard (*Anas platyrhynchos*) whose beak was maintained closed with a strip to avoid the consumption of gammarids. A net was added around the mesocosm to prevent the mallard to escape.

The number of infected and uninfected individuals at the air-water interface (i.e. between the water surface and 5-cm depth) was recorded at one, six, 24, 25, 48, 72, 91, 115 and 116 hours after their introduction. This was expected to reflect the amount of gammarids available for mallard predation. Time intervals were chosen so as to measure the short and long term behavioural responses of gammarids to changes in the biotic context (i.e. fish and mallard introduction). The mesocosms were checked regularly to remove and replace dead gammarids. All the fish survived and were returned to the sampling site after the experiment.

### Differential vulnerability to bird predation

First, predation tests were carried out in the mesocosms where we followed the vertical distribution of gammarids (Fig. 3). At the end of the mesocosm experiment (at 116 h), we allowed mallards to predate by removing the strip that closed their beak. Once 50 gammarids have been consumed, which took approximately 10 to 20 seconds, we stopped the experiment and counted the number of remaining individuals per infection status to calculate the Manly's preference index [29], [30], which accounts for prey depletion during the course of the experiment: *αi*  =  ln *Pp*/(ln *Pp* + ln *Ps*)

Where *αi* is the preference index for the chosen prey (here infected gammarids), *Pp* and *Ps* the proportion of infected and uninfected individuals left at the end of the experiment, respectively. The index ranges from zero (only uninfected prey consumed) to one (only infected prey consumed), and was compared to the theoretical value of 0.5 indicating the absence of differential predation between the two prey types. A differential predation with overconsumption of the infected prey would not provide evidence for predator preference but would be the consequence of a differential exposure to predation caused by infection. Second, additional predation tests were carried out to test if the differential predation observed in the mesocosms was due to the altered vertical distribution of infected gammarids only, or to other alterations such as colour changes. For instance, the use of painted mimics in a previous study suggested that *P. minutus*' colouration enhances the vulnerability to mallard predation [31]. Following the protocol described in [31], 54 infected and 54 uninfected *E. berilloni* were randomly distributed in 108 Petri dishes filled with 10 mL of water from the sampling site. This set-up standardizes the vertical repartition of infected and uninfected gammarids as the low water height (≈1 cm) does not allow any differential position in the water column [31]. The dishes were fixed on a dark green plate and the experiment took place in a four-m^2^ enclosure within the aviary to allow visual contacts between mallards and reduce stress (see [31]). Each mallard was introduced in the enclosure and allowed to predate until all dishes were empty, which took approximately 15 minutes. A camera (Logitech C910 HD pro Webcam) was fixed one m above the plate to record mallard's predation. We counted the number of infected and uninfected gammarids left once half of the available prey were eaten (i.e. 54) and calculated the Manly's *α* preference index to detect differential predation.

### Statistical analyses

To analyse the effect of infection status and olfactory context on the reaction to predation cue (proportion of time spent in the scented arm over the control arm), activity (total number of zones crossed divided by the time spent within the corresponding arm), and geotaxis and aggregation scores, we used Mixed Models with infection status, olfactory context (fish or mallard odour) and their interaction as fixed factors, and with the identity of the individual as random factor, to take into account the non-independance of measures taken on the same individual. When infection status was found to have an effect, then posthoc mixed models were conducted in each group (uninfected and infected). The normality and homosedasticity of residuals were checked for each model. To test whether gammarids displayed significant avoidance of predation cue and conspecific attraction, we compared the proportion of time spent in the scented arm and aggregation scores with neutral values (50% for avoidance, 0 for aggregation) using Wilcoxon paired tests.

It has been proposed that animal personality (consistent behavioural differences among individuals) and behavioural syndromes (correlations among behavioural traits) could be prime targets for host manipulation [32]. To test this assumption, we investigated the correlations between olfactory contexts for a given behaviour (personality), and correlations between behaviours for a given context (behavioural syndromes) using Spearman paired correlation tests with Bonferroni corrections.

To analyse the effect of infection status and fish presence on the vertical distribution of gammarids in the mesocosms, we used Generalized Mixed Models with the propotion of gammarids at the water surface versus in the rest of the mesocosm as a response variable and a quasibinomial distribution [33]. Infection status, time, experimental treatment (presence of fish or not), and their second-order interactions were included as fixed factors. The identity of the mesocosm was added as a random factor to account for the non-independance of gammarids from the same experimental unit (six replicates in each experimental group). Non significant terms were then removed sequentially to derive a minimal adequate model. A repeated ANOVA analysis was also performed to ensure the reliability of the results.

Finally, the differential vulnerability to bird predation of infected and uninfected gammarids was calculated using a Manly's index (see above) and compared with the neutral value of 0.5 using Wilcoxon tests. To test the effect of the presence of fish non-host predators on the predation by mallards, we ran a linear model with the Manly's index as response variable and fish treatment (presence or absence) as explanatory variable.

## Results

### Reaction to predation cue and activity

**Figure 4:**
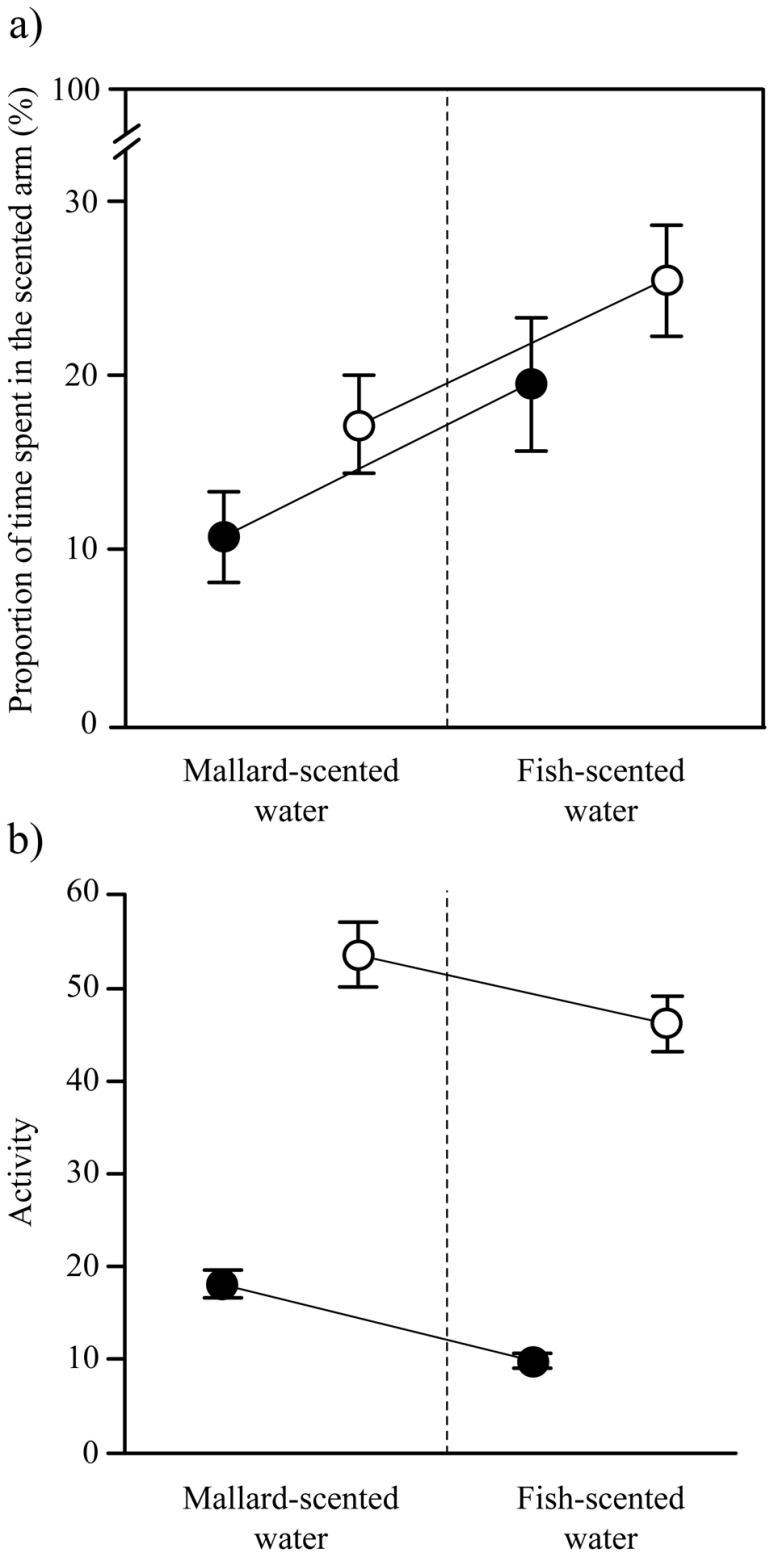
Reaction to predation cue (a) and activity (b) of *Echinogammarus berilloni* either uninfected (white dots) or infected with *Polymorphus minutus* (black dots). Reaction to predation cue (a) was measured as the proportion of time spent in the arm of an olfactometer scented with mallard (definitive host) or fish (non host) odour compared to the control arm (no scent). Activity (b) was measured as the number of areas visited in the whole olfactometer (scented and unscented arm) over 10 minutes. Values are means ± SD (N = 48 and 58 for infected and uninfected gammarids, respectively) and linked dots are paired data.

Both uninfected and infected gammarids significantly avoided the scented arm whatever the type of scent (Wilcoxon tests testing the difference with 50%: all P<0.001). However, they avoided the scent of mallard more than the scent of fish (Mixed model on the proportion of time spent in the scented arm with the individual as random effect, effect of odour: t_136_  = 2.01, P = 0.046, effect of infection status: t_138_ = 1.44, P = 0.15, interaction: t_136_ = −0.080, P = 0.94) (Fig. 4a). In addition, infected gammarids were significantly less active than uninfected ones (Mixed model on the number of zones crossed in the whole olfactometer, effect of infection status: t_138_ = 8.82, P<0.001, effect of odour: t_138_ = −7.12, P<0.001, interaction: t_138_ = 3.93, P<0.001) (Fig. 4b) Infected gammarids were significantly less active in the whole olfactometer when one of the arm was scented by fish odour compared to mallard odour (Posthoc test: Linear model, effect of odour in infected gammarids: estimate ± SE  = −0.64±0.093, t_69_ = −6.83, P<0.001), which was not the case for uninfected gammarids (Posthoc test: t_69_ = −1.64, P = 0.11). However, when analysing each arm separately and correcting for the time spent in each arm (i.e. number of zones crossed in one arm divided by the time spent in the corresponding arm), only infection status had an effect on activity, with infected individuals being less active than uninfected ones, whatever the olfactory context (Mixed model: effect of status: t_135_ = 3.09, P = 0.002).

### Geotaxis and conspecific attraction

**Figure 5:**
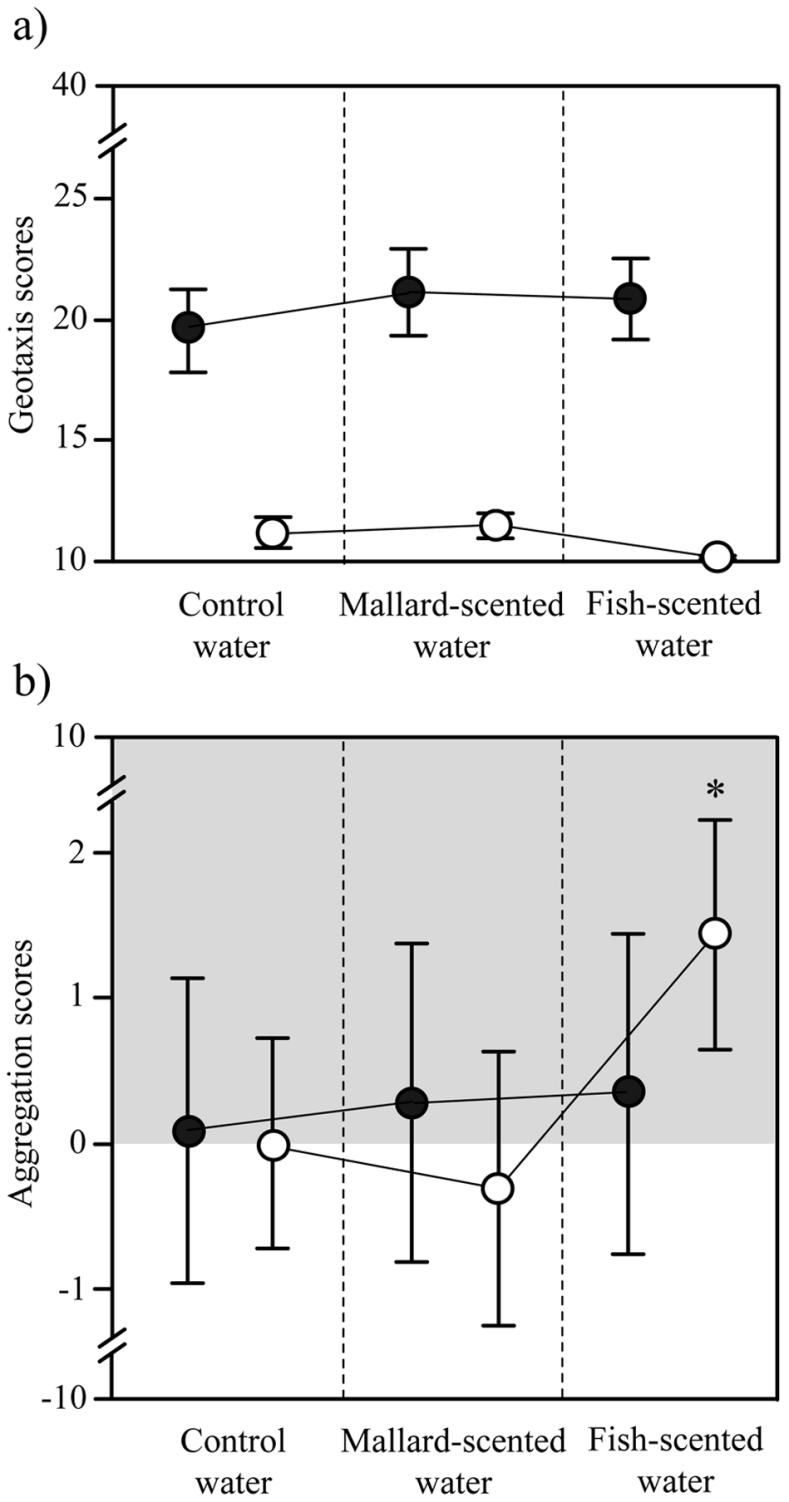
Geotaxis (a) and aggregation scores (b) of *Echinogammarus berilloni* either uninfected (white dots) or infected with *Polymorphus minutus* (black dots) successively tested in control (no predation cue), mallard-scented (definitive host cue) or fish-scented water (non-host cue). Values are means ± SD (N = 48 and 58 for infected and uninfected gammarids, respectively) and linked dots are paired data. The grey area indicates conspecific attraction.

Infected *E. berilloni* had a significantly higher geotaxis score (mean ± SE  = 20.5±0.99) than uninfected gammarids (mean ± SE  = 10.96±0.27) (Mixed model with a random effect of individual identity: effect of infection t_124_ = −8.07, P<0.001) (Fig. 5a). The olfactory context had no influence on geotaxis scores (effect of mallard scent: t_250_ = −0.89, P = 0.38, effect of fish scent: t_250_ = −0.84, P = 0.40) even in interaction with infection status (mallard scent x infection status: t_248_ = 0.63, P = 0.53; fish scent x infection status: t_248_ = −0.51, P = 0.61).

There was no significant effect of infection status or olfactory context on conspecific attraction (Mixed model, effect of infection status: t_124_ = 1.21, P = 0.23, olfactory context: t_248_ = 0.0034, P = 0.99, interaction: t_248_ = 0.45, P = 0.65) (Fig. 5b).

### Correlations across biotic contexts and between behaviours in microcosms

Only uninfected gammarids showed some degree of consistency in behaviours across olfactory contexts. Uninfected gammarids that were more active in fish-scented water were also more active in mallard-scented water (Paired Spearman correlation: ρ = 0.55, P<0.001), and uninfected gammarids that were more prone to aggregate in control water were also more prone to aggregate in mallard-scented water (ρ = 0.40, P<0.001). On the opposite, infected gammarids behaved unconsistently across contexts (all P values >0.12), except for geotaxis, which was high in both control and fish-scented water (ρ = 0.39, P = 0.016). No significant correlation between behaviours was found in infected and uninfected individuals (all P values >0.10 after Bonferroni correction).

### Vertical distribution in mesocosms

**Figure 6:**
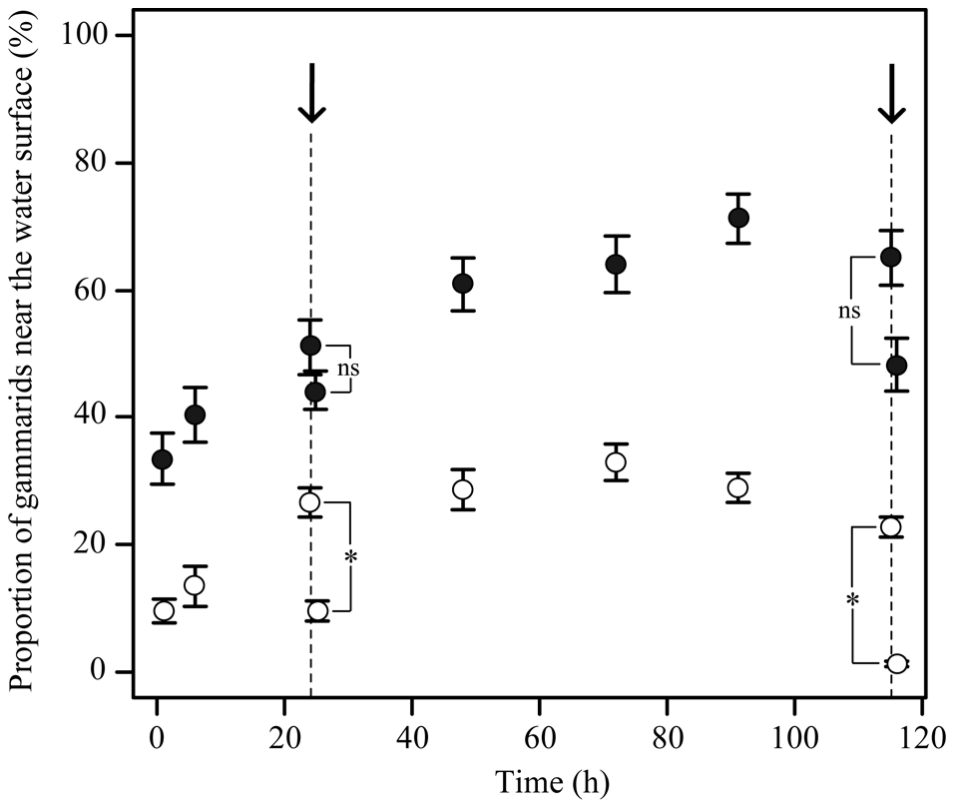
Proportions of *Echinogammarus berilloni* either uninfected (white dots) or infected with *Polymorphus minutus* (black dots) near the water surface followed over 120 h in mesocosms (see Fig. 1). The arrows indicate the successive introductions of a plastic tube (first arrow, at 24 h) and a mallard (second arrow, at 115 h) into each mesocosm. The plastic tube was either empty or filled with fish to mimic non-host predation risk. Because there was no significant difference in gammarids' distribution between the mesocosms with an empty tube or with a tube containing fish (see Table 1), we pooled all the experimental units in the figure. Values are means ± SD (N = 12 replicates). The asterisks show significant differences in the vertical distribution (ns for non significant).

The spatial repartition of gammarids in the mesocosms depended on the interaction between time and infection status, but not on the presence of fish in the mesocosms (Table 1). This shows that infected and uninfected gammarids had different behavioural responses to the introduction of tubes and mallards in the water (Fig 1), regardless of the presence of fish in the introduced tube. A repeated ANOVA analysis gave similar results (effect of infection status: F_1,258_ = 17.43, P<0.001; no effect of fish presence: F_1,257_ = 0.71, P = 0.40). At each recording time, the proportion of gammarids at the water surface was significantly higher for infected individuals (mean ± SE  = 0.49±0.016) compared to uninfected ones (mean ± SE  = 0.18±0.011) (Fig. 6). Posthoc tests show that the proportion of gammarids at the water surface decreased significantly after the successive introductions of the plastic tube (first arrow at 24 h, Fig. 6) (mean proportion before introduction ± SE  = 0.39±0.034; one hour after  = 0.27±0.039; paired Wilcoxon test: V = 272, P<0.001) and the mallard in each mesocosm (second arrow at 115 h) (mean proportion before introduction ± SE  = 0.44±0.049; one hour after: 0.25±0.056; paired Wilcoxon test: V = 249, P<0.001). The presence of fish inside the tube did not influence the behavioural response of gammarids to a change in their environment (i.e. tube and mallard introductions) (no effect of the fish presence x infection status interaction on gammarids' distribution, Table 1). Conversely, *P. minutus* infection significantly altered gammarids' response since the decrease in the proportion of individuals at the water surface was lower for infected gammarids than for uninfected gammarids (Fig. 6). Despite of this, the proportion of gammarids at the water surface remained significantly higher for infected gammarids compared to uninfected ones one hour after the introduction of the tubes (Mixed Model: effect of infection status: estimate ± SE  = −2.03±0.18, t_11_ = −11.06, P<0.001), or the mallards (estimate ± SE  = −0.47±0.042, t_10_ = −11.08, P<0.001) (Fig. 6).

**Table 1:** Best mixed model (with a random effect of the mesocosm identity) explaining the proportions of *Polymorphus minutus*-infected and uninfected gammarids at the water surface in the mesocosms.

	Estimate	DF	F	P
Time	0.0099±0.0016	247	6.18	<0.001
Infection status	−1.29±0.13	247	−9.97	<0.001
Time x Infection status	−0.0053±0.0021	247	−2.48	0.0137
Fish presence	-	-	-	-

The effect of fish presence in the tube did not affect the repartition of gammarids at the surface and was thus removed from the final model.

### Differential vulnerability to bird predation

**Figure 7:**
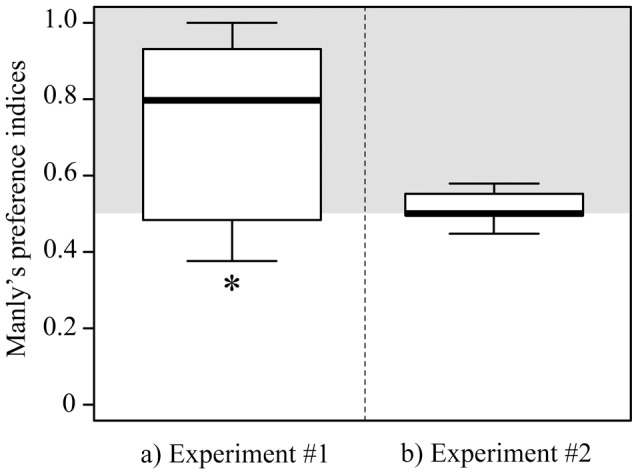
Manly's α preference index testing for a differential predation between *Echinogammarus berilloni* infected with *Polymorphus minutus* and uninfected gammarids in the presence of mallards. The predation of mallards was investigated twice: in the mesocosms where we followed the vertical distribution of gammarids (a), and with another experimental set up focusing on the difference in appearance between infected and uninfected gammarids (b) (see text for details). Values are medians and interquartile ranges (N = 12). The grey area indicates the overconsumption of infected gammarids and the asterisk shows a significant difference with the neutral value of 0.5 (no differential predation).

In the mesocosms where we followed gammarids' vertical distribution, infected individuals experienced a significantly higher predation by mallards than uninfected individuals (Wilcoxon test comparing the Manly's index to the value of 0.5: W = 108, P = 0.028) (Fig. 7a). This index was not influenced by the presence of fish in the mesocosm (Linear model, effect of fish treatment on preference index: t_10_ = 0.29, P = 0.77). In contrast, there was no such differential predation with the second set up standardizing the vertical distribution of infected and uninfected gammarids (Wilcoxon test: W = 97.5, P = 0.43) (Fig. 7b).

## Discussion

Host manipulation by parasites occurs in complex environments where both suitable and dead-end hosts coexist. Little is known on the role of such biotic context on parasite transmission. We first investigated to what extent the behavioural changes induced by *Polymorphus minutus* in its gammarid intermediate host were fine-tuned depending on whether the predator was a suitable host (mallard) or a non host (fish). We then investigated the trophic facilitation to mallard and tested whether it was influenced by the presence of non hosts.

In microcosms, both infected and uninfected gammarids showed repulsion to the scent of predator but avoided mallard odour more than fish odour, with no effect of infection. The difference in avoidance between the two predatory species might be due to the difficulty of standardizing the intensity of the two predation signals. Fish detection and avoidance is a widespread antipredator behaviour among invertebrates (e.g. [28]), which seems to be kept in *P. minutus* infected gammarids. This might benefit the parasite in terms of non-host predation risk [10]. Sometimes, host manipulation consists in turning repulsion into attraction when the predator is a suitable definitive host [26], [34], [35]. We found no such evidence of attraction to definitive hosts, as infected gammarids avoided mallard odour as well as uninfected individuals. However, this did not prevent the trophic transmission to mallards, as shown by the overconsumption of infected gammarids observed during predation tests.

In agreement with the literature, infection with *P. minutus* significantly reduced gammarids' activity [10], [36]. The decrease was more important with fish cues than with mallard cues, which might limit detection and predation by non-host fish and therefore could be interpreted as a protective behaviour induced by the parasite [27], [37]. However, this should be taken with caution since the same trend was found in uninfected gammarids. This suggests that the behaviour of infected gammarids does not depend on the type of predator.

Another result suggesting that there is no fine-tuning of manipulation by *P. minutus* is that the olfactory context had no effect on the geotaxis and aggregation scores of infected gammarids. They never aggregated and were always found at the water surface in the microcosms and mesocosms whatever the biotic context. In another study, the aggregation behaviour of *G pulex* was not suppressed by *P. minutus* [36]. This discrepancy between results may be due to differences in host exploitation between the two gammarid species or differences in the experimental design since Thünken *et al*. [36] did not account for geotaxis reversion. Together with predator avoidance and reduced activity, conspecific attraction is reported as another antipredatory response that reduces predation risk through a dilution effect [27], [38]. Infected gammarids being at the top of the water column, the suppression of conspecific attraction is likely to increase predation risk by surface-dwelling predators, including waterbird definitive hosts, and could therefore be considered an adaptive manipulation. However, the contribution of aggregation suppression relative to the other parasite-induced changes (i.e. geotaxis reversion) to the increased trophic transmission to definitive hosts remains to be tested.

Taken together, the results of the microcosm experiments suggest that rather than turning off the whole antipredator strategy, infection disrupts some behavioural responses (positive geotaxis, conspecific attraction) but not others (predator avoidance, reduced activity). Interestingly, only uninfected gammarids showed some degree of consistency in behavioural scores across olfactory contexts, while we found no such correlation in infected individuals (except for geotaxis that was consistently high). This is in accordance with previous studies showing that infection with a trematode reduces the repeatability of behaviour in amphipods [39], which might cause inappropriate behavioural responses to environmental stimuli [32]. We found however no evidence for an effect of the parasite on syndrome structure (strength of relationships between behavioural traits) as suggested in previous studies [39], [40]. These findings support the growing literature showing that parasitism has the potential to affect animal so-called personality traits [41], [42], which might be particularly relevant for manipulative parasites, since affecting the consistency and relationships between traits could yield greater benefits than affecting single traits or only their mean values [40], [43]. A fruitful avenue of research will be to determine the role of infection-driven variability in personality traits on transmission probability and ultimately on parasite fitness. In addition, it is possible that differential environmental exposure to predators and/or selective predation on certain behavioural types (the most active gammarids for instance) of the wild-caught gammarids tested here might shape part of the variance in behaviour in this study. Experiments on lab-raised gammarids would be useful to compare the adaptive value of environmental versus genetic-based behavioural variability in wild populations regarding predation risk and parasite transmission.

In another set of experiments, we examined how the presence of non-host and definitive host predators influences the vertical distribution of gammarids and ultimately parasite's transmission. In the mesocosms mimicking natural conditions, the proportion of infected gammarids found at the air-water interface always exceeded that of uninfected individuals, even after several days. This is in line with the geotaxis reversion observed in microcosms, but inconsistent with a previous study [21] showing that *P. minutus*-infected *Gammarus roeseli* were as benthic as uninfected gammarids over the long term. In Médoc *et al*. [21], food source and refugia were provided at the bottom of the experimental units only, which might have encouraged infected gammarids to stay close to the bottom after several hours. Following the introduction of either plastic tubes or mallards in the mesocosms, most uninfected gammarids reached the bottom while only a limited fraction of infected individuals reacted in the same way. Sheltering in benthic substrates is part of the antipredator strategy of gammarids and seems to be disrupted by *P. minutus* infection, which is likely to explain the overconsumption of infected gammarids by mallards. The presence of fish in plastic tubes did not affect gammarids' vertical distribution, whatever their infection status. Although we chose fish and gammarid densities so as to have approximately the same number of predation events than in microcosms, the resulting fish predation signal may have been too low to influence the behaviour of gammarids in mesocosms. On the other hand, this could be consistent with the idea that geotaxis reversion is a permanent state, displayed whatever the biotic context, rather than a short-term response triggered by environmental cues.

The mechanisms underpinning such behavioural alterations remain however elusive. A higher oxygen need for infected gammarids is unlikely to explain geotaxis reversion as infection with *P. minutus* has been shown to lower oxygen consumption in *Gammarus roeseli* [44]. Similar results have been found in *G. pulex* infected with *Pomphorhynchus laevis* [45]. In addition, Médoc *et al*. [46] found no evidence for neutral lipid depletion in *P. minutus*-infected *G. roeseli*, which, compared to uninfected gammarids, had a higher concentration of triglycerides and displayed better swimming performances [9]. This discards the possibility that *P. minutus*-induced alterations result from an energetic drain of infection, at least at the cystacanth stage. Alternatively, alterations of the neuromodulatory system and of the synthesis of monoamine neurotransmitters might underlie the observed differences in activity, movements and social activity between infected and uninfected gammarids [47]. For instance, an hemocoel injection of serotonin (5-HT) into uninfected gammarids was found to elicit the phototaxis reversion displayed by *G. pulex* infected with either *Pomphorhynchus laevis* or *P. terreticolis* [48]. Results are less clear concerning geotaxis reversion, which is not triggered by 5-HT injection in *G. pulex* [48], but induced by a long-term exposure to 5-HT in *Echinogammarus marinus* [49]. Recently, Helluy [50] suggested that the inflammation of the central nervous system caused by hemocelian parasites such as acanthocephalans could account for the alterations of the sensorimotors pathways observed in manipulated gammarids. A methodological approach combining phenotypic engineering with a screening of host's transcriptome or proteome and parasite's secretome would help us to fully identify the physiological bases of behavioural manipulation [51].

Whatever the underlying mechanisms explaining behavioural changes, they induced a differential predation by mallards with an overconsumption of infected gammarids compared to uninfected gammarids, which provide, to our knowledge, the first experimental evidence of the trophic facilitation induced by *P. minutus*. No such differential predation was observed in controlled conditions standardizing gammarids' vertical repartition, suggesting that the enhanced trophic transmission observed in mesocosms was mainly due to the altered vertical distribution associated with geotaxis reversion. A recent experiment conducted with the same set-up showed that mallards significantly preferred orange-painted *E. berilloni* (mimicking infected prey) over brown-painted gammarids (mimicking uninfected prey) [31]. The discrepancy between the two studies suggests that using paint to mimic *P. minutus* infection does not fully reproduce the parasite-induced change in appearance and overestimates its role on trophic transmission. Similar results were obtained with the fish acanthocephalan *Pomphorhynchus laevis*: mimicking colour changes in uninfected gammarids did not increase their vulnerability to definitive hosts, which always preferred manipulated individuals even when the parasite was hidden [52]. The present study brings additional support to the idea that the carotenoid-based colouration of acanthocephalans has no adaptive value in terms of transmission, or at least a minor role compared to reversed geotaxis. It also suggests that reversed geotaxis is the central trait enhancing transmission, and that the presence of non hosts in the environment has no effect on enhanced transmission

Previous findings on the *P. minutus*-gammarid system led us to consider a quite sophisticated scenario of manipulation with a possible role for non hosts in triggering the phenotypic alterations that facilitate trophic transmission [10], [21]. The present study brings support for a more parsimonious explanation of manipulation associating a chronic dimension through geotaxis reversion and reduced activity, and a phasic dimension with altered reactions to chemical (no conspecific attraction) or physical cues (no sheltering at the bottom), which could all result from parasite-induced alterations of sensorimotor pathways. Given the wide dispersal range of waterbirds, the definitive hosts of *P. minutus*, such manipulation that is effective (i.e. through the expression of parasite-induced behavioural changes) regardless of the biotic context could facilitate trophic transmission in a wide range of aquatic environments compared to a more fine-tuned manipulation that would work better in particular environments but less well in all others [53]. Although experimental work is still needed to test this hypothesis, the reduced vulnerability to non hosts reported in previous studies may thus not be specifically selected for but be a beneficial by-product of a non-specific behavioural alteration enhancing predation. The alterations underlying predation suppression might also have evolved to accommodate predation risk in aquatic communities when the ancestors of *P. minutus* reproduced in invertebrates and before the inclusion of aquatic birds as second and definitive host *via* upward incorporation [54], [55].

## References

[1] Moore J (2002) Parasites and the behavior of animals. 1st ed. Oxford University Press, USA. 338 p.

[2] Lefèvre T, Roche B, Poulin R, Hurd H, Renaud F, et al (2008) Exploiting host compensatory responses: the “must” of manipulation? Trends Parasitol 24: 435–439 10.1016/j.pt.2008.06.006 18707919

[3] Poulin R (1995) “Adaptive” changes in the behaviour of parasitized animals: A critical review. Int J Parasitol 25: 1371–1383 10.1016/0020-7519(95)00100-X 8719948

[4] Poulin R (2010) Parasite manipulation of host behavior. Advances in the Study of Behavior. Elsevier, Vol. 41 . pp. 151–186. Available: http://linkinghub.elsevier.com/retrieve/pii/S0065345410410050. Accessed 29 October 2012.

[5] Maure F, Payette Daoust S, Brodeur J, Mitta G, Thomas F (2013) Diversity and evolution of bodyguard manipulation. J Exp Biol 216: 36–42 10.1242/jeb.073130 23225865

[6] Hammerschmidt K, Koch K, Milinski M, Chubb JC, Parker GA (2009) When to go: optimization of host switching in parasites with complex life cycles. Evolution 63: 1976–1986 10.1111/j.1558-5646.2009.00687.x 19453381

[7] Anderson RA, Koellaf JC, Hurd H (1999) The effect of *Plasmodium yoelii nigeriensis* infection on the feeding persistence of *Anopheles stephensi* Liston throughout the sporogonic cycle. Proc R Soc Lond B Biol Sci 266: 1729–1733. 10.1098/rspb.1999.0839 PMC1690202 10518321

[8] Dianne L, Perrot-Minnot M-J, Bauer A, Gaillard M, Léger E, et al (2011) Protection first then facilitation: a manipulative parasite modulates the vulnerability to predation of its intermediate host according to its own developmental stage. Evol Int J Org Evol 65: 2692–2698 10.1111/j.1558-5646.2011.01330.x 21884065

[9] Médoc V, Beisel J-N (2008) An acanthocephalan parasite boosts the escape performance of its intermediate host facing non-host predators. Parasitology 135. Available: http://www.journals.cambridge.org/abstract_S0031182008004447. Accessed 24 October 2012. 10.1017/S0031182008004447 18477417

[10] Médoc V, Rigaud T, Bollache L, Beisel J-N (2009) A manipulative parasite increasing an antipredator response decreases its vulnerability to a nonhost predator. Anim Behav 77: 1235–1241 10.1016/j.anbehav.2009.01.029

[11] Cézilly F, Grégoire A, Bertin A (2000) Conflict between co-occurring manipulative parasites? An experimental study of the joint influence of two acanthocephalan parasites on the behaviour of *Gammarus pulex* . Parasitology 120: 625–630. 10874725 10.1017/s0031182099005910

[12] Bauer A, Haine ER, Perrot-Minnot M-J, Rigaud T (2005) The acanthocephalan parasite *Polymorphus minutus* alters the geotactic and clinging behaviours of two sympatric amphipod hosts: the native *Gammarus pulex* and the invasive *Gammarus roeseli* . J Zool 267: 39 10.1017/S0952836905007223

[13] Mouritsen KN, Poulin R (2003) Parasite-induced trophic facilitation exploited by a non-host predator: a manipulator's nightmare. Int J Parasitol 33: 1043–1050. 13129526 10.1016/s0020-7519(03)00178-4

[14] Lagrue C, Kaldonski N, Perrot-Minnot MJ, Motreuil S, Bollache L (2007) Modification of host's behavior by a parasite: field evidence for adaptive manipulation. Ecology 88: 2839–2847. 18051653 10.1890/06-2105.1

[15] Cézilly F, Perrot-Minnot M-J (2005) Studying adaptive changes in the behaviour of infected hosts: a long and winding road. Behav Processes 68: 223–228 10.1016/j.beproc.2004.08.013 15792694

[16] Médoc V, Beisel J-N (2011) When trophically-transmitted parasites combine predation enhancement with predation suppression to optimize their transmission. Oikos 120: 1452–1458 10.1111/j.1600-0706.2011.19585.x

[17] Seppala O, Valtonen ET, Benesh DP (2008) Host manipulation by parasites in the world of dead-end predators: adaptation to enhance transmission? Proc R Soc B Biol Sci 275: 1611–1615 10.1098/rspb.2008.0152 PMC2602814 18430644

[18] Parker GA, Ball MA, Chubb JC, Hammerschmidt K, Milinski M (2009) When should a trophically transmitted parasite manipulate its host? Evolution 63: 448–458 10.1111/j.1558-5646.2008.00565.x 19154358

[19] Ness JH, Foster SA (1999) Parasite-associated phenotype modifications in threespine stickleback. Oikos 85: 127 10.2307/3546798

[20] Kaldonski N, Perrot-Minnot M-J, Motreuil S, Cézilly F (2008) Infection with acanthocephalans increases the vulnerability of *Gammarus pulex* (Crustacea, Amphipoda) to non-host invertebrate predators. Parasitology 135. Available: http://www.journals.cambridge.org/abstract_S003118200800423X. Accessed 11 October 2012. 10.1017/S003118200800423X 18371238

[21] Médoc V, Bollache L, Beisel J-N (2006) Host manipulation of a freshwater crustacean (*Gammarus roeseli*) by an acanthocephalan parasite (*Polymorphus minutus*) in a biological invasion context. Int J Parasitol 36: 1351–1358 10.1016/j.ijpara.2006.07.001 16934814

[22] Médoc V, Beisel J-N (2009) Field evidence for non-host predator avoidance in a manipulated amphipod. Naturwissenschaften 96: 513–523 10.1007/s00114-008-0503-8 19139837

[23] Cézilly F, Perrot-Minnot M-J (2010) Interpreting multidimensionality in parasite-induced phenotypic alterations: panselectionism versus parsimony. Oikos 119: 1224–1229 10.1111/j.1600-0706.2010.18579.x

[24] Thomas F, Poulin R, Brodeur J (2010) Infection syndrome and multidimensionality: two terms for two different issues. Oikos 119: 1230–1230 10.1111/j.1600-0706.2010.18975.x

[25] Kaldonski N, Perrot-Minnot M-J, Cézilly F (2007) Differential influence of two acanthocephalan parasites on the antipredator behaviour of their common intermediate host. Anim Behav 74: 1311–1317 10.1016/j.anbehav.2007.02.027

[26] Perrot-Minnot M-J, Kaldonski N, Cézilly F (2007) Increased susceptibility to predation and altered anti-predator behaviour in an acanthocephalan-infected amphipod. Int J Parasitol 37: 645–651 10.1016/j.ijpara.2006.12.005 17258219

[27] Durieux R, Rigaud T, Médoc V (2012) Parasite-induced suppression of aggregation under predation risk in a freshwater amphipod: sociality of infected amphipods. Behav Processes 91: 207–213 10.1016/j.beproc.2012.08.002 22940109

[28] Wudkevich K, Wisenden BD, Chivers DP, Smith RJF (1997) Reactions of *Gammarus lacustris* to chemical stimuli from natural predators and injured conspecifics. J Chem Ecol 23: 1163–1173 10.1023/B:JOEC.0000006393.92013.36

[29] Manly BFJ (1974) A model for certain types of selection experiments. Biometrics 30: 281–294 10.2307/2529649

[30] Chesson J (1983) The estimation and analysis of preference and its relationship to foraging models. Ecology: 1297–1304.

[31] Jacquin L, Mori Q, Médoc V (2013) Does the carotenoid-based colouration of *Polymorphus minutus* facilitate its trophic transmission to definitive hosts? Parasitology: 1–6. 10.1017/S0031182013000760 23866854

[32] Poulin R (2013) Parasite manipulation of host personality and behavioural syndromes. J Exp Biol 216: 18–26 10.1242/jeb.073353 23225863

[33] Zuur A, Ieno EN, Saveliev AA, Smith GM (2009) Mixed effects models and extensions in Ecology with R. Springer. 580 p.

[34] Berdoy M, Webster JP, Macdonald DW (2000) Fatal attraction in rats infected with *Toxoplasma gondii* . Proc R Soc Lond B Biol Sci 267: 1591–1594 10.1098/rspb.2000.1182 PMC1690701 11007336

[35] Baldauf SA, Thünken T, Frommen JG, Bakker TCM, Heupel O, et al (2007) Infection with an acanthocephalan manipulates an amphipod's reaction to a fish predator's odours. Int J Parasitol 37: 61–65 10.1016/j.ijpara.2006.09.003 17049528

[36] Thünken T, Baldauf SA, Bersau N, Bakker TCM, Kullmann H, et al (2010) Impact of olfactory non-host predator cues on aggregation behaviour and activity in *Polymorphus minutus* infected *Gammarus pulex* . Hydrobiologia 654: 137–145 10.1007/s10750-010-0377-6

[37] Bollache L, Kaldonski N, Troussard J-P, Lagrue C, Rigaud T (2006) Spines and behaviour as defences against fish predators in an invasive freshwater amphipod. Anim Behav 72: 627–633 10.1016/j.anbehav.2005.11.020

[38] Evans SR, Finnie M, Manica A (2007) Shoaling preferences in decapod crustacea. Anim Behav 74: 1691–1696 10.1016/j.anbehav.2007.03.017

[39] Coats J, Poulin R, Nakagawa S (2010) The consequences of parasitic infections for host behavioural correlations and repeatability. Behaviour 147: 367–382 10.1163/000579509X12574307194101

[40] Hammond-Tooke CA, Nakagawa S, Poulin R (2012) Parasitism and behavioural syndromes in the fish *Gobiomorphus cotidianus* Available: http://www.otago.ac.nz/parasitegroup/PDF%20papers/Hammond-Tookeetal2012-Behav.pdf. Accessed 20 September 2012.

[41] Kortet R, Hedrick AV, Vainikka A (2010) Parasitism, predation and the evolution of animal personalities. Ecol Lett 13: 1449–1458 10.1111/j.1461-0248.2010.01536.x 21040352

[42] Barber I, Dingemanse NJ (2010) Parasitism and the evolutionary ecology of animal personality. Philos Trans R Soc B Biol Sci 365: 4077–4088 10.1098/rstb.2010.0182 PMC2992744 21078659

[43] Cézilly F, Favrat A, Perrot-Minnot M-J (2013) Multidimensionality in parasite-induced phenotypic alterations: ultimate versus proximate aspects. J Exp Biol 216: 27–35 10.1242/jeb.074005 23225864

[44] Lukacsovics F (1959) *Polymorphus minutus* Goeze (Acanthocephala) Larva hatasa a *Gammarus roeseli* Gerv. (Amphipoda) fajra. Ann Inst Biol (Tihany) Hung Acad Sci 26: 31–39.

[45] Rumpus AE, Kennedy CR (1974) The effect of the acanthocephalan *Pomphorhynchus laevis* upon the respiration of its intermediate host, *Gammarus pulex* . Parasitology 68: 271–284 10.1017/S0031182000045789 4826717

[46] Médoc V, Piscart C, Maazouzi C, Simon L, Beisel J-N (2011) Parasite-induced changes in the diet of a freshwater amphipod: field and laboratory evidence. Parasitology 138: 537–546 10.1017/S0031182010001617 21232173

[47] Lafferty KD, Shaw JC (2013) Comparing mechanisms of host manipulation across host and parasite taxa. J Exp Biol 216: 56–66 10.1242/jeb.073668 23225868

[48] Tain L, Perrot-Minnot M-J, Cezilly F (2006) Altered host behaviour and brain serotonergic activity caused by acanthocephalans: evidence for specificity. Proc R Soc B Biol Sci 273: 3039–3045 10.1098/rspb.2006.3618 PMC1679890 17015346

[49] Guler Y, Ford AT (2010) Anti-depressants make amphipods see the light. Aquat Toxicol Amst Neth 99: 397–404 10.1016/j.aquatox.2010.05.019 20591511

[50] Helluy S (2013) Parasite-induced alterations of sensorimotor pathways in gammarids: collateral damage of neuroinflammation? J Exp Biol 216: 67–77. 23225869 10.1242/jeb.073213

[51] Perrot-Minnot M-J, Cézilly F (2013) Investigating candidate neuromodulatory systems underlying parasitic manipulation: concepts, limitations and prospects. J Exp Biol 216: 134–141 10.1242/jeb.074146 23225876

[52] Kaldonski N, Perrot-Minnot M-J, Dodet R, Martinaud G, Cezilly F (2009) Carotenoid-based colour of acanthocephalan cystacanths plays no role in host manipulation. Proc R Soc B Biol Sci 276: 169–176 10.1098/rspb.2008.0798 PMC2614247 18796399

[53] Seppälä O (2008) Jokela (2008) Host manipulation as a parasite transmission strategy when manipulation is exploited by non-host predators. Biol Lett 4: 663–666. 18700200 10.1098/rsbl.2008.0335 PMC2614144

[54] Beisel J-N, Médoc V (2010) Bird and amphipod parasites illustrate a gradient from adaptation to exaptation in complex life cycle. Ethol Ecol Evol 22: 265–270.

[55] Cézilly F, Thomas F, Médoc V, Perrot-Minnot M-J (2010) Host-manipulation by parasites with complex life cycles: adaptive or not? Trends Parasitol 26: 311–317 10.1016/j.pt.2010.03.009 20392669

